# Guidelines for Sport Compressive Garments Design: Finite Element Simulations Approach

**DOI:** 10.3390/muscles4040042

**Published:** 2025-10-09

**Authors:** Alessandro Cudicio, Marta Cogliati, Gianluca Rizzi

**Affiliations:** 1 Department of Human and Social Sciences, University of Bergamo, 24129 Bergamo, Italy; 2 Department of Clinical and Experimental Sciences, University of Brescia, 25123 Brescia, Italy; marta.cogliati@unibs.it; 3 Chair of Continuum Mechanics, Faculty of Architecture and Civil Engineering, TU-Dortmund, August-Schmidt-Str. 8, 44227 Dortmund, Germany; gianluca.rizzi@tu-dortmund.de

**Keywords:** compressive garments, finite element simulation, customized garment design, graduated compression, sport garment, sport performance

## Abstract

Purpose: Despite significant attention being paid to compression garments (CG) in the sports field, there remains ongoing debate regarding their actual effectiveness in enhancing athletic performance and expediting post-exercise recovery. This article examines their various aspects, with a focus on CG design and the materials they are made of, aiming to analyze the importance of personalized compression strategies based on individual anthropometric measurements and non-linear compression designs. Methods: Using anthropometric analysis of 40 healthy participants, this study examines the morphological characteristics of the lower limb and their implications for CG design. Results: Measurements of limb length and circumferences revealed complex interactions among anatomical variables, emphasizing the need for customized and adaptable device design. Finite element simulations clarified the challenges in achieving uniform pressure gradients along the lower limb, highlighting the limitations of one-piece devices and suggesting tailored segmented designs for individual limb segments. Conclusion: The results demonstrate that one-piece devices often fail to provide optimal compression due to non-linear variations in limb dimensions. Conversely, segmented devices, particularly those with bilinear progression, exhibited superior performance in applying targeted compression across different limb segments. This more detailed approach to customization could significantly contribute to optimizing outcomes and user comfort.

## 1. Introduction

Compression garments (CG) have emerged as a focal point in the world of sports, finding widespread use across various disciplines, be they individual- or team-oriented. Their significance lies in the diverse range of potential effects they offer, primarily centered around two key aspects: performance enhancement and expedited recovery for athletes [1,2]. CG, mostly used on the lower limbs, are supposed to be purposefully crafted to apply targeted pressure to specific areas with the overarching goal of optimizing blood circulation, giving muscle support, and improving overall athletic performance. The demands placed on CG vary not only with the specific sport, but also with the timing and modality of their application [3,4,5]. In the realm of sports science, CG has garnered considerable attention as a promising tool for athletes seeking a competitive edge [2,6]. By enhancing venous blood flow [7] and mitigating muscle vibration [8] during and after physical activities, CG holds the potential to augment muscle performance and endurance. Moreover, the post-exercise application of CG has been linked to reduced muscle soreness [1,9] and faster recovery times [2], making them useful for athletes who want to refine their training regimens and maximizing their potential [1] or for non-athletes who want to reduce post-exercise symptoms.

During physical activity, the use of CG could promote an increase in the production of nitric oxide (NO) [10]. When released, NO acts as a chemical signal that induces relaxation of the smooth muscle cells present in the walls of blood vessels, allowing for them to dilate [10]. It has been shown that intermittent pneumatic compression not only affects venous blood flow in the compressed area, but also produces systemic effects on circulation. Variations in central venous pressure, pulmonary artery pressure, and pulse pressure have been identified thanks to the increase in venous return induced by leg compression [11,12]. Additionally, intermittent pneumatic compression of the foot has been found to have significant local effects on microcirculation, increasing blood flow [13]. The diameter of arterial and venous vessels in distant skeletal muscles during intermittent pneumatic compression suggests increased NO release; this vasodilatory effect improves microcirculation and its effects on physical activity [7].

Similarly, CGs have been used for their effects on microcirculation and other systems. Indeed, the application of CG has been long-established in therapeutic medicine for the prevention and management of various conditions such as lymphedema, pulmonary embolism, deep vein thrombosis, wounds, scars, and venous leg ulcers [1]. Subsequently, the use of compression has been extended to the field of sports, with the introduction of CG marketed as a tool to improve various aspects of physical performance. While some evidence suggests that CG can reduce muscle oscillation, improve joint awareness, influence oxygen use during low-intensity exercise, modify local blood flow, reduce swelling, and alleviate muscle pain during post-exercise recovery, scientific consensus on their performance-enhancing effects in different forms of exercise remains limited [1]. Studies have analyzed the effects of compression on hemodynamic parameters, such as blood vessel diameter and blood flow, as well as on athletic performance and the reduction in muscle fatigue [1]. This ongoing debate is partly fueled by the limitations of commercially available, ‘ready-to-use’ garments, which fail to account for individual anthropometric variability and may not provide optimal pressure.

Determining the optimal compression level for different muscle groups is a complex but important process, with recommended pressures varying depending on the muscle group. For instance, pressure on the leg should be 17 mmHg, while pressure on the thigh should be 15 mmHg [14]; high compression levels can alter blood flow, whereas low compression levels may prove ineffective. However, the effectiveness of ready-to-use commercially available compression garments may be affected by the lack of information on individual body dimensions, leading to the use of generalized sizing systems that do not adequately account for the variability in body shapes and sizes [14].

While studies have shown promising results regarding the positive effects of CG on muscle recovery, the effectiveness may vary based on factors such as duration of usage, applied pressure, and the type of exercise performed [15]. It is essential to evaluate these variables and consider that the technical characteristics of compression can represent a key factor for efficient compression.

The aim of this study is to establish a design methodology for lower limb CGs that maximize their potential benefits by analyzing the morphological characteristics of the leg and thigh, and integrating these findings into finite element simulations to optimize garment design and material selection. Through a collaborative effort between sports scientists and materials engineers, we seek to advance knowledge in this area, providing valuable insights to optimize CG production and their use during training and recovery strategies.

## 2. Materials and Methods

### 2.1. Subjects

Forty healthy participants (20 males and 20 females; age: 21 ± 2.9 YO; height: 172 ± 9.2 cm; weight: 66 ± 2.9 kg) were enrolled in this study. Inclusion criteria required participants to be in good health and maintain at least a moderate level of physical activity. Furthermore, this research adhered to the ethical guidelines outlined in the Declaration of Helsinki. Prior to participation, all individuals read and provided their signature on an informed consent form that had been approved by the local Ethics Committee (protocol number 5108). The sample size was estimated using G*Power 3.1.9.7 for a correlation, and bivariate normal model and input variables (tails = 2, correlation p H1 = 0.60, alpha  =  0.05, power  =  0.98) were estimated. The estimated minimum sample size was 36.

### 2.2. Lower Limb Measurements

The limb chosen for measurement was the dominant leg of each subject. A measuring tape designed for nutritionists (RENPHO, Irvine, CA, USA) was used to measure the circumference and length of the lower limb. To be specific, measurements were recorded at the proximal, medial, and distal thirds of both the thigh and the leg. The lower limb’s length was determined by measuring from the greater trochanter as the starting point to the malleolus as the endpoint. For a detailed description of the measurement procedure, see Tan et al., 2013 [16].

Lower limb measurements served two primary objectives: first, to describe the correlations between the size and length of the leg and thigh segments to assess the sustainability of current sizing systems; and second, to provide realistic anthropometric data for the finite element simulations.

### 2.3. Simulation

**Figure 1:**
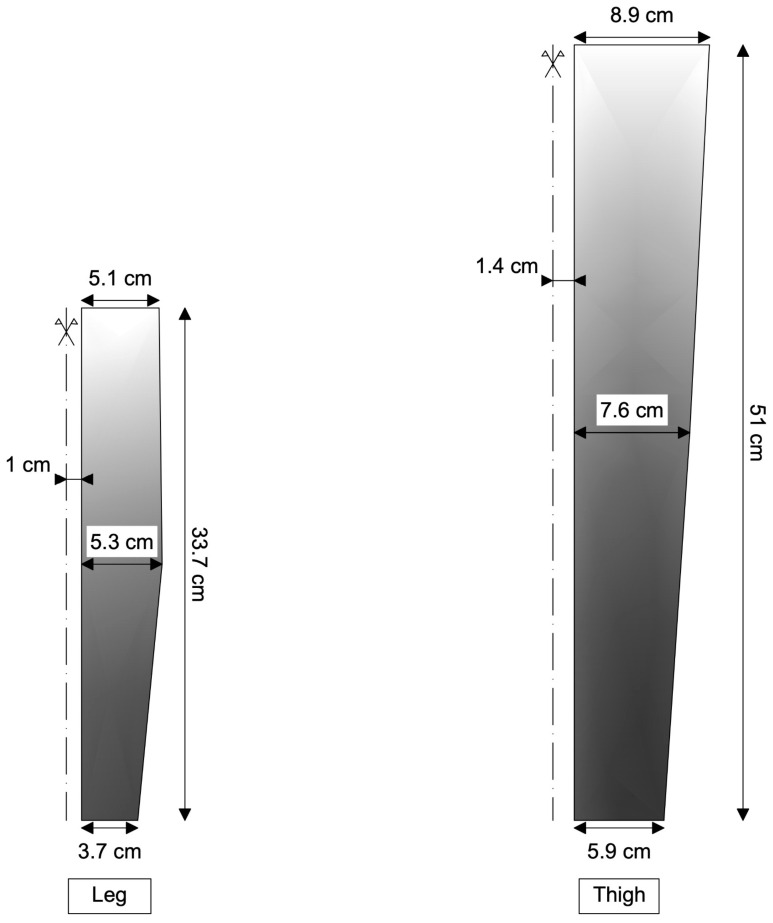
Lower limb: Schematics and measures of the lower limb built with axial-symmetric symmetry. The latter has been divided into leg and thigh, while both of them have been further divided into two sub-parts of equal size linearly varying along the length according to [ref measurement].

Finite element simulations [17,18,19] were conducted using a simplified model of the lower limb to investigate how the geometry and materials used in compression garments affect their performance. The lower limb has been modeled with axial-symmetric symmetry. The dimensions for the simulated lower limb (Figure 1) were derived from the average anthropometric measurements, previously described, ensuring the model’s anatomical realism. While this representation may not capture all of the intricate three-dimensional anatomical complexities, this simplification is justified for an exploratory approach, as it allows for efficient isolation and analysis of the fundamental mechanical principles governing pressure distribution in compression garments along the longitudinal axis. This approach significantly reduces computational complexity from a full 3D problem to a more manageable 2D one (Figure 1), enabling a focused investigation into the impact of garment design and material progression on pressure gradients, which is the primary aim of this study.

The geometry of the lower limb is portrait in Figure 1. The limb has been divided into leg and thigh, and both of them have been further divided into two sub-parts of equal size whose thickness linearly varies along the length. This simplified geometry, while more streamlined, still accounts for the essential features. Both the leg and the thigh have been modeled as being made up of bones and muscles (see Singh et al., 2021 [20], and Table 2 for the elastic parameters), since it is assumed that the contribution of the other tissues is negligible.

The bones are considered as cylinders with a radius of 1 cm for the leg and 1.4 cm for the thigh, as shown in Figure 1. Since the bones are much stiffer than the soft tissues, they are considered infinitely stiff. This allows for omitting their modeling and instead clamping the muscle tissue with an offset relative to the axis of axial symmetry.

The geometry of the compression garments has been modeled in two different ways:

(A) One lower limb garment with a linear progression;
(B) Two independent garments for the leg and the thigh, with a linear progression;
(C) Two independent garments for the leg and the thigh, with a bilinear progression;

In Table 1, we provide measures for the proximal, central, and distal radii of the CG for the case (C), while for cases (A) and (B), also listed in Table 1, only the proximal and distal radii are included.

The CG has been modeled with two materials, nylon and a sparse fabric made up of 75% Nylon and 25% Elastan (Elastam25).

The values of the elastic parameters for the nylon have been taken directly from the COMSOL Multiphysics^®^ (v6.0) software library (the suite was also used to perform the simulations), while the Young’s modulus of the sparse fabric was taken from [21]. Given the unavailability of a specific Poisson’s ratio for the sparse fabric in the literature, we will consider the Poisson’s ratios for its two constituents and obtain the one for the spacer fabric using the well-established rule of mixtures [22]. The Poisson’s ratio for nylon typically ranges from 0.25 to 0.4 [23,24], while for elastane, it typically ranges from 0.40 to 0.5 [25]. Given the percentage in Table 2, the rule of mixtures gives a range from 0.28 to 0.42. The mean value chosen is 0.35. However, a sensitivity analysis has been conducted by introducing two additional Poisson’s ratio values for the sparse fabric, specifically 0.25 and 0.45.

These values resulted in a constant variation in pressure along the limb section under consideration, with a maximum increase of +1 mmHg for 0.45 and a maximum decrease of −1 mmHg for 0.25. This variation is negligible for the scope of the present study.

All of the material parameters are summarized in Table 2.

**Figure 2:**
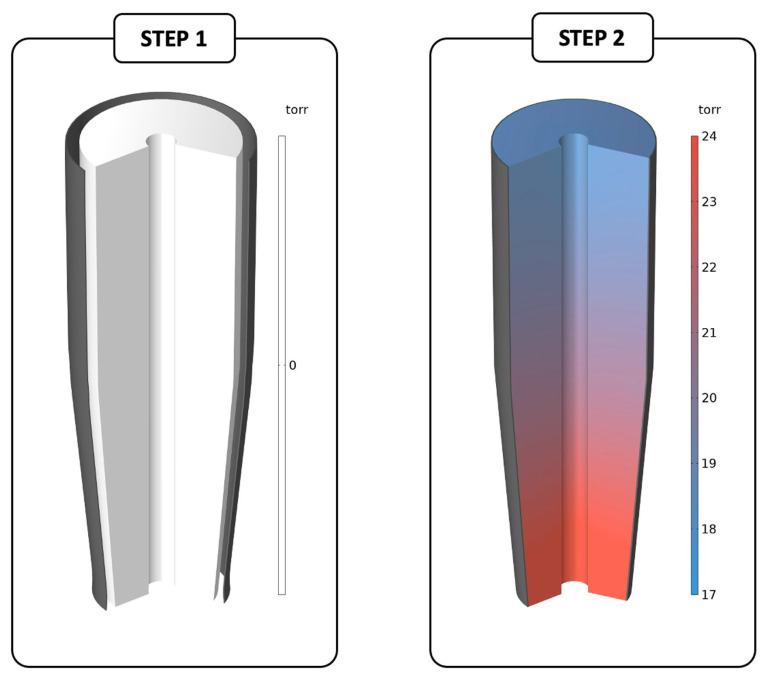
Exemplary steps of the simulation (3D geometry). (**left**) During the first step, the compressive garment is stretched because of a load that is applied to its outer surface; (**right**) during the second step, the load is gradually reduced until it is completely removed, while the contact condition on the black boundaries is enforced.

**Figure 3:**
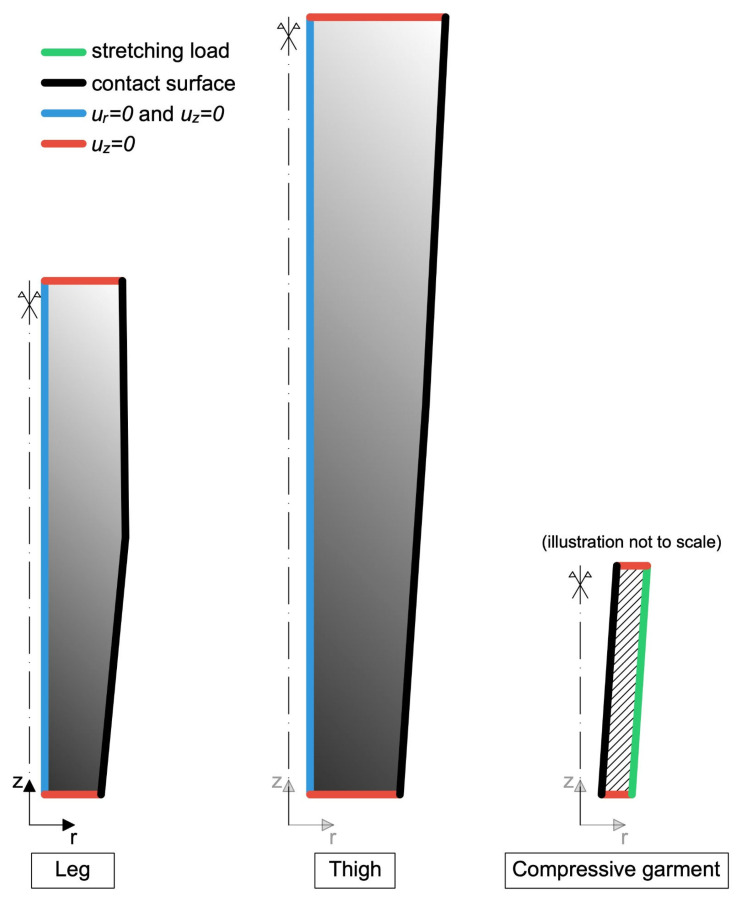
Lower limb boundary conditions. The blue boundaries have been fully clamped, which means that it has been chosen ur = 0 (displacement component along the r axis) and uz = 0 (displacement component along the *z*-axis); on the red boundaries, the displacement along the *z*-axis has been chosen uz = 0; on the black boundaries, a monolateral contact condition with the compressive garments has been enforced (co-penetration is not permitted, but detachment is allowed); on the green boundary, a load is applied in order to stretch the compressive garment.

All of the simulations were carried out using COMSOL Multiphysics^®^ software (Structural Mechanics Module), and comprise two steps (Figure 2):

(1) The CG is initially simulated independently, and a load is applied along the positive r direction on the green boundary (Figure 3) to stretch the garment beyond the size of the lower limb;
(2) Following that, the lower limb is introduced, the contact constraint on the black boundaries is enforced between the two bodies (Figure 3), and, gradually, the load is reduced until it is completely removed.

The boundary conditions are represented in Figure 3, and are as follows:

- The boundaries in blue have been fully clamped in the r and z directions (ur = 0 and uz = 0);
- On the boundaries in red, it is prescribed uz = 0;
- On the boundaries in black, a monolateral frictionless contact condition with the CG has been enforced (co-penetration is not permitted, but detachment is allowed);
- On the boundary in green, a load is applied in order to stretch the CG (step 1), and it is then gradually decreased until it is completely removed (step 2).

**Figure 4:**
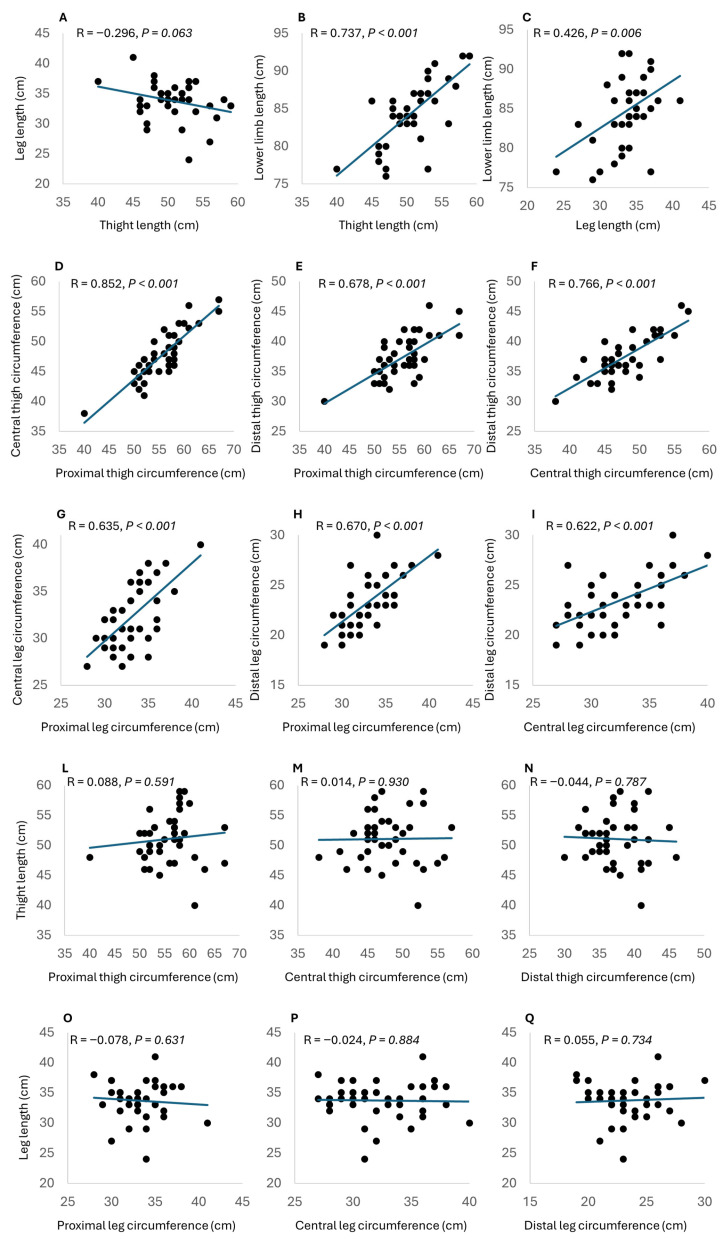
Panels (**A**–**C**): Scatter plots for the relationship (black dots) between leg length and thigh length (**A**), lower limb length and thigh length (**B**), and lower limb length and leg length (**C**). The correlation between the variables is represented by the regression line (blue line). Panels (**D**–**F**): Scatter plots for the relationship (black dots) between central thigh circumference and proximal thigh circumference (**D**), distal thigh circumference and proximal thigh circumference (**E**), and distal thigh circumference and central thigh circumference (**F**). The correlation between the variables is represented by the regression line (blue line). Panels (**G**–**I**): Scatter plots for the relationship (black dots) between central leg circumference and proximal leg circumference (**G**), distal leg circumference and proximal leg circumference (**H**), and distal leg circumference and central leg circumference (**I**). The correlation between the variables is represented by the regression line (blue line). Panels (**L**–**N**): Scatter plots for the relationship (black dots) between thigh length and proximal thigh circumference (**L**), central thigh circumference (**M**), and distal thigh circumference (**N**). The correlation between the variables is represented by the regression line (blue line). Panels (**O**–**Q**): Scatter plots for the relationship (black dots) between leg length and proximal leg circumference (**O**), central leg circumference (**P**), and distal leg circumference (**Q**). The correlation between the variables is represented by the regression line (blue line).

**Figure 5:**
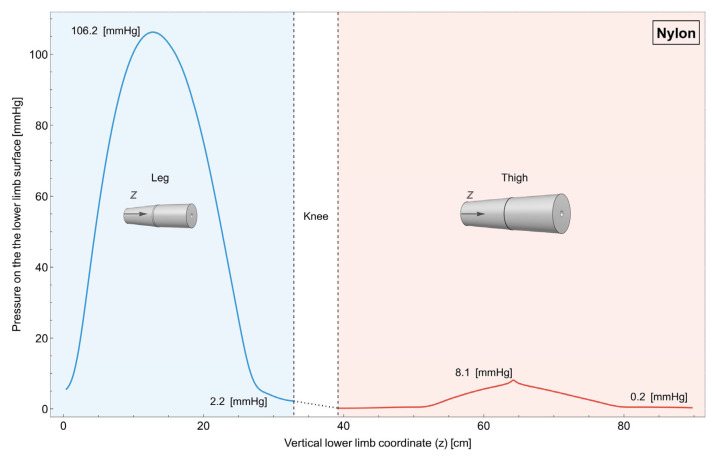
Lower limb garment made of nylon (single piece). Variation in pressure on the surface of the lower limb along its length. It can be observed that, with a single garment, it is not possible to obtain a uniformly decreasing pressure starting from the distal part of the leg and arriving to the proximal part of the thigh.

**Figure 6:**
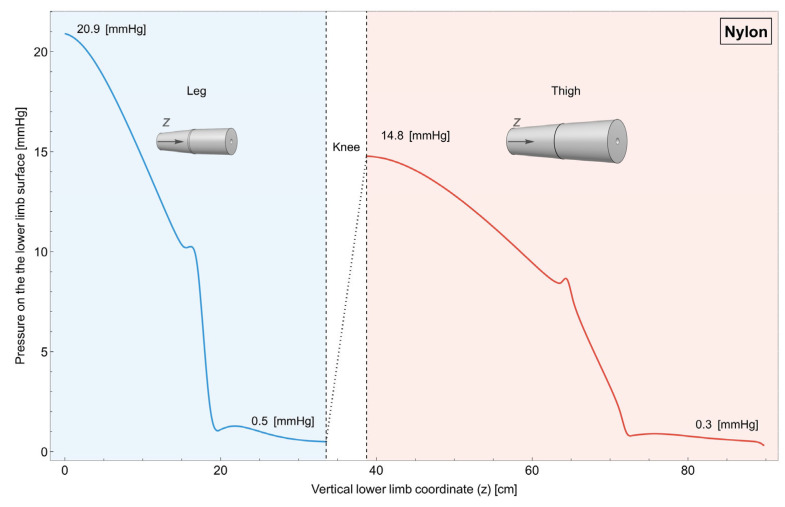
Individual linear progression garment made of nylon. Variation in pressure on the surface of the lower limb along its length. It can be observed that it is possible to tune the pressure in the distal part of both the leg and the thigh while keeping a decreasing value for the pressure. In order to keep reasonable pressure in the distal part, the contact between the garment and the proximal part is lost.

### 2.4. Statistical Analysis

Statistical analysis was performed using Jamovi for Windows, version 2.3.23. To determine statistical significance, a type I error rate of 0.05 was employed. Pearson’s correlation coefficient was utilized to examine potential interactions among the lengths of the lower limb, leg, and thigh, as well as the circumferences of the proximal medial and distal portions of the leg and thigh. Given that the lower limb measurement’s primary objective is descriptive and that covariates (such as age or body composition) are not typically considered in garment manufacturing, we limited our analysis to Pearson’s correlations to assess the fundamental relationships between key variables.

## 3. Results

Measurements were recorded at the proximal, medial, and distal thirds of both the thigh and the leg, with the lower limb’s length measurements. These results are presented in Table 3 for easy reference.

In the Pearson correlation chart, the relationship between the total length of the lower limb and the lengths of the thigh and leg can be observed (Figure 4A–C). The data highlights a significant correlation between lower limb length and both thigh length (r = 0.737, *p* < 0.001) and leg length (r = 0.426, *p* = 0.006), indicating a strong association between these variables. However, no statistical correlation was found between the leg and the thigh length (r = −0.296; *p* = 0.063).

### 3.1. Lower Limb Girth

In this investigation, we observed notable distinctions in thigh circumferences when measured at various anatomical positions (Figure 4D–F). Specifically, the correlation analysis revealed significant relationships between these circumferences. The central high circumference exhibited a robust positive correlation with proximal thigh circumference (r = 0.852, *p* < 0.001), underscoring a strong association between these two measurements. Similarly, a significant positive correlation was observed between medial thigh circumference and distal thigh circumference (r = 0.766, *p* < 0.001), indicating a substantial connection between these variables. Furthermore, the distal thigh circumference displayed a significant positive correlation with proximal thigh circumference (r = 0.678, *p* < 0.001).

In this comprehensive analysis, significant variations in leg circumferences when measured at distinct anatomical positions were also uncovered (Figure 4G–I). Our correlation analysis reveals robust and statistically significant relationships among these circumference measurements. Specifically, the central leg circumference exhibited a noteworthy positive correlation with distal leg circumference (r = 0.622, *p* < 0.001), highlighting a substantial connection between these two variables. Additionally, a significant positive correlation was observed between central leg circumference and proximal leg circumference (r = 0.635, *p* < 0.001), indicating a strong association between these measurements. Furthermore, the distal leg circumference demonstrated a significant positive correlation with proximal leg circumference (r = 0.670, *p* < 0.001)

### 3.2. Lower Limb Length and Girth

The correlation analysis between thigh length and thigh circumferences yielded non-significant results (Figure 4L–N). Specifically, at the proximal level, the correlation coefficient was r = 0.109 (*p* = 0.502), at the medial level it was r = 0.102 (*p* = 0.536), and at the distal level it was r = −0.044 (*p* = 0.787).

Similarly, the correlation analysis between leg circumferences measured at corresponding points and leg length revealed non-significant findings (Figure 4O–Q).

Specifically, at the proximal level, the correlation coefficient was r = −0.078 (*p* = 0.631), at the medial level it was r = −0.024 (*p* = 0.884), and at the distal level it was r = 0.055 (*p* = 0.734).

### 3.3. Simulation

**Figure 7:**
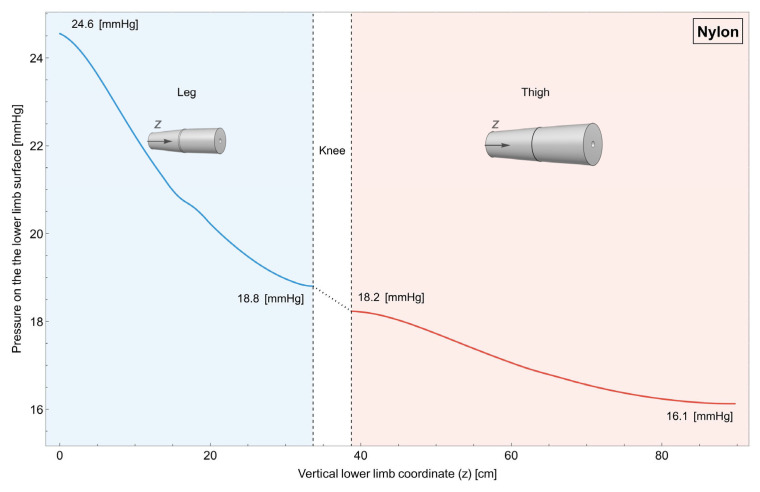
Individual bilinear progression garments nylon. Variation in the pressure on the surface of the lower limb along its length. It can be observed that it is possible to tune the pressure in both the distal and proximal part of both the leg and the thigh while keeping a decreasing value for the pressure.

**Figure 8:**
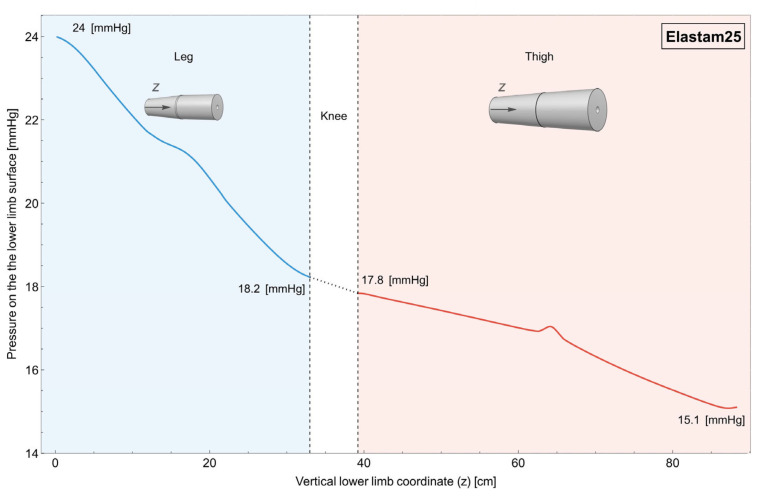
Individual bilinear progression garments Elastam25. Variation in pressure on the surface of the lower limb along its length. It can be observed that it is possible to tune the pressure in both the distal and proximal part of both the leg and the thigh while keeping a decreasing value for the pressure.

In Figure 5, Figure 6, Figure 7 and Figure 8, we report how the pressure on the surface of the lower limb changes along its length while changing the type of garment.

In Figure 5, it is reported how the pressure changes on the surface of the lower limb for the case in which the garment is a single piece made of nylon.

As can be seen, achieving a consistently decreasing pressure gradient from the distal end of the leg to the proximal end of the thigh is not feasible. This is due to the fact that the radius of the leg does not change linearly along its length, which makes the outermost part the most compressed.

In order to overcome this limitation, two individual linear garments are proposed.

In Figure 6, it is possible to observe how the pressure varies when two individual garments for the leg and the thigh made of nylon are used. For this case, the radius of the garments changes linearly along their length and independently between the two.

As can be seen, it is possible to fine-tune pressure levels in the distal regions of both the leg and thigh, maintaining a decrease in pressure. However, to maintain reasonable pressure in the distal areas, contact between the garment and the proximal part needs to be sacrificed, and this is not desirable, especially in the proximal part of the leg.

To overcome this drop in pressure close to the proximal areas, two individual bilinear garments are here proposed.

In Figure 7, it is possible to observe the case in which the garments are made of nylon, while in Figure 8, it is possible to observe the case in which the garments are made of Elastam25 (75% nylon–25% elastane).

In both of these cases, the radius of the garments changes bilinearly along their length and independently between the two.

As can be seen, it is now possible to both tune the value of the pressure in the distal and proximal regions of both the leg and thigh, while maintaining a decrease in pressure.

## 4. Discussion

In our study, the morphological characteristics of the lower limb underscore the complexity of designing CG. The existing and missing significant correlations between the limb measurements suggest that a one-size-fits-all approach may not be suitable. This finding aligns with previous research highlighting the importance of individualized garment designs tailored to athletes’ unique anatomies [1,26,27,28]. Research consistently shows the importance of personalizing physical activity from didactic [29], physiological [30], and psychological [31] perspectives. Tailoring the tools used for physical activity or sport can significantly enhance performance. Moreover, our simulation results shed light on the challenges in achieving a uniformly decreasing pressure gradient along the limb’s length. Comparing our findings with existing literature [32] it becomes evident that the key lies not only in the choice of materials but also in the garment’s design [33]. Unlike previous studies [33] our simulations considered both linear and bilinear progressions, revealing nuanced pressure variations across different garment types.

### 4.1. Optimal Compression

The impacts of compression in the sports science field are widely acknowledged. Nonetheless, the effectiveness of compression is conditioned by its quantity and quality, as these factors determine the intended outcome. Incorrect application of compression can potentially negate its desired effects. Specifically, in the context of physical activity, a minimum compression of 15 mmHg [14,34,35,36] is deemed necessary for an effective outcome. This threshold cannot be guarantee with linear compressive models, as can be seen in Figure 5 and Figure 6. Moreover, excessively high compression, like in the case of the leg in the single-piece garment (Figure 5), can be counterproductive, potentially restricting blood flow and causing discomfort. Moreover, the ideal compression entails a graduated increase in pressure distally, diminishing gradually in a proximal direction [36,37,38]. Individual bilinear progression garments, both using nylon and Elastam25, guarantee this progression (Figure 7 and Figure 8).

The variation in leg dimensions, including circumference and length (Figure 4), highlights the crucial need to account for these individual morphological traits when applying compression. The insufficient or ineffective compression observed in certain cases, as noted from the previous paper from Hill et al. 2015 [14], may stem from attempts to standardize garment sizes. This practice might inherently be deemed a mistake, as it overlooks the diverse anatomical features among individuals, potentially resulting in inadequate compression for some.

### 4.2. Length and Circumference of the Limb

The results of this study unveil a notable correlation between the total length of the lower limb and both thigh and leg lengths. Interestingly, however, no significant correlation emerged between thigh length and leg length, a finding of particular relevance in CG design. This discrepancy underscores a critical factor in garment design efficiency.

To ensure effective compression of the entire lower limb, the garment must encompass the entire limb [14,34]. The absence of a correlation between thigh length and leg length (Figure 4A) highlights the need for a more adaptable approach to garment design. To achieve optimal results, the garment’s length must be dynamically adjusted to accommodate various combinations of thigh and leg segment lengths. Additionally, significant correlations were observed among the proximal, central, and distal circumferences within both segments (Figure 4D–F for the thigh and Figure 4G–I for the leg). This underscores that the width of the CG can be categorized uniformly. However, no correlations were identified between any circumference and its corresponding segment length (Figure 4L–N for the thigh and Figure 4O–Q for the leg), indicating that the design of CG must meticulously consider both of these variables. This insight underscores the need for adaptable, custom-made designs in CG manufacturing. These designs should accommodate the distinct yet interconnected aspects, taking into account the need to design CG of varying lengths for different widths.

Our anthropometric findings, particularly the lack of correlation between leg and thigh lengths and between length and circumference, directly informed our investigation into different garment designs. These findings highlighted the inherent limitations of single-piece linearly progressing garments, and motivated the exploration of segmented and bilinear designs in our simulations to better accommodate individual limb variability.

### 4.3. Simulation

Compression, based on anatomical observations, must be carefully calibrated to avoid a linear increase from the ankle to the thigh. This necessity arises from observed data indicating a non-linear pressure increase. This trend can be better interpreted as the intersection of two lines with different inclinations for each individual leg segment. A targeted compression approach is essential for proper support and optimal blood circulation in every leg region. This customization considers the specific anatomical needs of each individual [28,39,40], adapting to variations in leg conformation. Maintaining pressure that respects these anatomical characteristics significantly contributes to overall well-being, avoiding the inefficacy of uniform compression and allowing for the better management of each individual’s physiological peculiarities.

In order to assess how the design of different garments would affect the pressure distribution along the lower limb, three options have been simulated using finite element analysis.

#### 4.3.1. Single Linear Garments

The results from our simulation of a single linear garment (see Figure 5) indicate that this design is unable to generate sufficient and effective compression from the lower part of the leg to the upper part of the thigh. These variations, namely the non-uniform changes in limb dimensions along their length, impact the single garment’s ability to apply adequate pressure uniformly. While single garments may offer greater comfort during use [36], they present significant limitations in delivering the expected benefits of graduated compression. To address this limitation, it is necessary to produce a range of garments with diverse ergonomic features that can accommodate the varying morphological characteristics of users. Consequently, the number of templates which take into account the length and circumferences of the lower limb segments may prove to be too extensive to be economically sustainable.

#### 4.3.2. Separated Linear Garment

In second instance, the use of two separate linear garments is proposed: one dedicated to the thigh, and the other to the lower leg. This configuration allows for a more customized application of compression, adjusting pressure levels differentially to accommodate the specific anatomical requirements of each leg part. We proceeded with a detailed analysis of the effects resulting from the use of these two separate garments, distinguishing between possible outcomes when using two distinct linear garments or two separate bilinear garments. Typically, the design of individual garments is linear, as simpler techniques are required for production. As shown in Figure 6, the results for the individual linear CG indicate that desired pressure values can be achieved in the distal portion of both the thigh and the lower leg. However, it is important to note that, although the intensity of the average pressure on the skin may be considered sufficient, challenges arise related to the gradient and quality of the pressure, with a significant cost observed in the proximal part of the limb (~0.3–0.5 mmHg), resulting in a loss of pressure. This outcome uncovers two potential issues associated with the application of two distinct linear CG applied separately on the leg and thigh. Firstly, the reduced pressure in the proximal areas might not yield the anticipated physiological benefits due to an insufficient amount of pressure [14]. Secondly, the increased compression in the distal part of the thigh, compared to the proximal part of the leg, could result in a “funnel effect” that may counteract the intended facilitation of venous return [7,34].

#### 4.3.3. Separated Bilinear Garment

Finally, in addressing these challenges, the use of two separate bilinear CGs was examined, as shown in Figure 7 (nylon) and Figure 8 (Elastam25). This design allows for a more targeted application of compression with the ability to adjust pressure levels differentially for the thigh and lower leg, ensuring optimal compression and tangible benefits. The minimum pressure, obtained by the simulation on the upper part of the thigh, varies around 15–16 mmHg, depending on the material set, ensuring that the minimal pressure has a positive effect on local hemodynamics [14]. However, the higher pressure, recorded on the distal part of the leg, does not exceed values that could be counterproductive [41]. Furthermore, the proximal gradual reduction in the pressure may efficiently aid venous return, reducing blood stagnation. This bilinear approach emerges as a more promising solution to achieve maximum benefits in terms of blood circulation and muscle support. In the case of two separate bilinear garments, regardless of the material they are made of (nylon or Elastam25), optimal compression is achieved both distally and proximally while maintaining a gradual decrease in pressure from the lower limb’s base to its upper region. This promotes the attainment of the anticipated benefits [2,7,15] from using these garments.

### 4.4. Material

Nylon is a commonly used synthetic material for creating durable, lightweight fabrics [42,43]. It is renowned for its resistance to wear, abrasion, and moisture. Elastane, on the other hand, is a common material used in fabric production. It is a synthetic fiber known for its remarkable elasticity and ability to return to its original shape [44]. The presence of elastane in fabrics allows for them to better conform to the body and maintain their original shape, even after use and washing. When comparing these two materials, it is important to note their distinct qualities [41]. Each material serves specific purposes in the field of textiles, with nylon offering strength and longevity, while elastane provides comfort and adaptability to body contours. This is the reason why we have chosen to include both of these materials in the present study.

However, a direct comparison of Figure 7 and Figure 8 highlight that, while both materials with a bilinear design achieve the desired pressure gradient, the Elastam25 garment appears to offer a more uniform and consistent decrease in pressure, likely due to its superior elasticity. This leads to the hypothesis of better performance in terms of blood circulation, muscle function, and post-exercise recovery. An essential factor favoring Elastam25 garments is also the material they are composed of.

### 4.5. Limitations and Perspectives

The present study shows some limitations: (1) Only two types of materials were considered. While the market offers a wide range of materials, we chose to focus on the most commonly used in the construction of compressive fabrics. However, it is important to acknowledge that we cannot generalize the conclusions drawn from our findings to all types of compressive fabrics, although we can do this for a wide range of commercial materials, as long as they can be considered homogeneous. Still, future research could explore a more diverse range of materials to provide a more comprehensive understanding of how different fabric compositions affect the performance of CG. (2) The simulation was conducted on a two-dimensional model, whereas considering the morphology of the leg in three dimensions would be more representative of real-world conditions. However, we know that the morphology of the leg is much more intricate, with curves, contours, and variations in volume that cannot be accurately captured in two dimensions. Future studies should aim to incorporate three-dimensional models to better account for the anatomical complexities and provide more realistic assessments of CG performance. (3) The present study is based on simulations and data analysis to identify the ideal characteristics of a compressive fabric. However, no tests have been conducted on human subjects to verify the effectiveness of the compression proposed in the simulation. Consequently, an important area of future development would be to translate the concept of compression into prototypes of real compressive fabrics and conduct tests to evaluate the effectiveness of such fabrics on human subjects.

## 5. Conclusions

This study’s analysis of lower limb morphology reveals that leg and thigh dimensions vary independently, making a one-size-fits-all approach to CG design ineffective for maximizing their potential benefits in athletic performance and recovery. Our finite element simulations confirmed this by showing that conventional one-piece garments fail to provide the desired graduated compression along the limb.

The most effective solution proposed is a segmented design using two separate bilinear garments for the leg and thigh. This approach successfully applies targeted, graduated pressure, ensuring optimal compression in both distal and proximal regions. This performance was consistent across different material types, providing a clear design principle for future garments and directly addressing the limitations of current standardized products.

In essence, this research establishes a new methodology for CG design. By combining individual anthropometric data with a bilinear, segmented garment design, it is possible to achieve superior, more comfortable compression, which will likely lead to improved compliance and effectiveness for the end-user. Based on the simulation results, subsequent experiments should employ compressive garments divided into two segments, each characterized by a bilinear profile. These findings provide a solid foundation for the development of physical prototypes and further in vivo testing to validate this innovative approach.

## Figures and Tables

**Table 1:** Compressive garments: Values of the radii at midpoint for the compressive garments.

			Radius at Midpoint [cm]		
Case	Material	Kind of Garment	Proximal	Central	Distal
(A)	Nylon	single (lower limb)	9.0	-	3.8
(B)	Nylon	linear (leg)	6.5	-	3.7
		linear (thigh)	9.3	-	5.9
(C)	Nylon	bilinear (leg)	5.3	5.1	3.7
		bilinear (thigh)	8.9	7.6	5.9
	Sparse fabric—Elastam25 (75% nylon–25% elastane)	bilinear (leg)	4.5	4.2	3.1
		bilinear (thigh)	7.2	6.1	4.9

**Table 2:** Values of the material parameters of the muscles and the fabric composing the compression bands.

	Young’s Modulus [Pa]	Poisson’s Ratio [-]
Muscle	0.03 × 10^6^	0.48
Nylon	2000 × 10^6^	0.4
Sparse fabric—Elastam25 (75% nylon–25% elastane)	0.24 × 10^6^	0.35

**Table 3:** Average values of the measurements of the dominant leg of each participant. Measurements were recorded at the proximal, medial, and distal thirds of both the thigh and the leg. The lower limb’s length was determined by measuring from the greater trochanter as the starting point to the malleolus as the endpoint.

Length (cm)			Thigh Circumference (cm)			Leg Circumference (cm)		
Thigh	Leg	Lower Limb	Proximal	Medial	Distal	Proximal	Medial	Distal
51.07 ± 4.12	33.7 ± 3.08	84.75 ± 4.36	55.67 ± 4.88	47.75 ± 4.11	37.35 ± 3.52	33.20 ± 2.61	32.35 ± 3.43	23.42 ± 2.57

## Data Availability

The raw data supporting the conclusions of this article are available from the corresponding author upon reasonable request.

## Abbreviations

The following abbreviations are used in this manuscript:

CG	Compression Garments
NO	Nitric Oxide
